# Changes in upper airways microbiota in ventilator-associated pneumonia

**DOI:** 10.1186/s40635-023-00496-5

**Published:** 2023-03-02

**Authors:** Laura Alagna, Leonardo Mancabelli, Federico Magni, Liliane Chatenoud, Gabriele Bassi, Silvia Del Bianco, Roberto Fumagalli, Francesca Turroni, Davide Mangioni, Guglielmo M. Migliorino, Christian Milani, Antonio Muscatello, Giovanni Nattino, Edoardo Picetti, Riccardo Pinciroli, Sandra Rossi, Tommaso Tonetti, Alessia Vargiolu, Alessandra Bandera, Marco Ventura, Giuseppe Citerio, Andrea Gori

**Affiliations:** 1 Present Address: Infectious Diseases Unit, Department of Internal Medicine, Fondazione IRCCS Ca’ Granda Ospedale Maggiore Policlinico, Milan, Italy; 2 Department of Medicine and Surgery, University of Parma, Parma, Italy; 3 Department of Chemistry, Life Sciences and Environmental Sustainability, University of Parma, Parma, Italy; 4 Neurointensive Care Unit, Fondazione IRCCS San Gerardo dei Tintori, Monza, Italy; 5 Istituto di Ricerche Farmacologiche Mario Negri IRCCS, Milan, Italy; 6 Intensive Care, ASST GOM Niguarda, Milan, Italy; 7 School of Medicine and Surgery, University of Milano-Bicocca, Milan, Italy; 8 Interdepartmental Research Centre Microbiome Research Hub, University of Parma, Parma, Italy; 9 Department of Pathophysiology and Transplantation, University of Milan, Milan, Italy; 10 Infectious Diseases Unit, Fondazione IRCCS San Gerardo dei Tintori, Monza, Italy; 11 Department of Anesthesia and Intensive Care, Parma University Hospital, Parma, Italy

**Keywords:** Ventilator-associated pneumonia, VAP, Upper airways microbiota, 16S-rRNA microbial profiling, Cohort study, Mechanical ventilation

## Abstract

**Background:**

The role of upper airways microbiota and its association with ventilator-associated pneumonia (VAP) development in mechanically ventilated (MV) patients is unclear. Taking advantage of data collected in a prospective study aimed to assess the composition and over-time variation of upper airway microbiota in patients MV for non-pulmonary reasons, we describe upper airway microbiota characteristics among VAP and NO-VAP patients.

**Methods:**

Exploratory analysis of data collected in a prospective observational study on patients intubated for non-pulmonary conditions. Microbiota analysis (trough 16S-rRNA gene profiling) was performed on endotracheal aspirates (at intubation, T0, and after 72 h, T3) of patients with VAP (cases cohort) and a subgroup of NO-VAP patients (control cohort, matched according to total intubation time).

**Results:**

Samples from 13 VAP patients and 22 NO-VAP matched controls were analyzed. At intubation (T0), patients with VAP revealed a significantly lower microbial complexity of the microbiota of the upper airways compared to NO-VAP controls (alpha diversity index of 84 ± 37 and 160 ± 102, in VAP and NO_VAP group, respectively, *p*-value < 0.012). Furthermore, an overall decrease in microbial diversity was observed in both groups at T3 as compared to T0. At T3, a loss of some genera (*Prevotella* 7, *Fusobacterium*, *Neisseria*, *Escherichia–Shigella* and *Haemophilus*) was found in VAP patients. In contrast, eight genera belonging to the Bacteroidetes, Firmicutes and Fusobacteria phyla was predominant in this group. However, it is unclear whether VAP caused dysbiosis or dysbiosis caused VAP.

**Conclusions:**

In a small sample size of intubated patients, microbial diversity at intubation was less in patients with VAP compared to patients without VAP.

**Supplementary Information:**

The online version contains supplementary material available at 10.1186/s40635-023-00496-5.

## Background

Microbiota is the ecological community of commensal and symbiotic organisms that inhabit a specific body space and interact with local physiological mechanisms [1]. Recent significant advances in respiratory research and the implementation of next-generation sequencing technologies debunked the old dogma of lung sterility [2]. According to experimental models, the gut microbiota can influence the pulmonary microbiota, which confirms the existence of a microbiota gut–lung axis via blood or lymphatic translocation from the altered gut mucosa permeability to the lung [3, 4].

Literature reported an abundance in the lung microbiota composition of *Prevotella*, *Streptococcus*, *Veillonella*, *Fusobacterium* and *Haemophilus,* but this can be highly variable [5]*.*

Physiological homeostasis among commensals residing in the lungs is obtained through a specific and complex mechanism. It has been suggested that the migration of bacteria from the upper respiratory tract is balanced by active microbial elimination, allowing the selection of favorable microorganisms that will indeed constitute the microbiota of healthy lungs [6].

According to studies, a stable commensal ecosystem is a key feature of a healthy microbiota. However, the equilibrium can be distorted by several distinct events defined as perturbations [7], such as pathologies altering natural host defences [8–10], infections, diet or drug intake [8, 11]. A specific feature of the microbiota is resilience, defined as the ability of the microbial community to recover its initial function or taxonomical composition following accentuated perturbation [7, 12, 13].

Mechanical ventilation (MV) is one condition that aggressively interferes with physiological lung homeostasis [14]. Recent studies have reported that lung microbiota undergoes a profound reduction in the diversity of species in all MV patients [15–17]. To date, most of the studies refer to MV patients as a whole, with no distinction on the reasons that lead to intubation (i.e., pulmonary or non-pulmonary conditions) [17–19]. However, this distinction could be important, especially when analyzing the dynamics of pulmonary microbiota and the potential association with MV-related events such as ventilator-associated pneumonia (VAP).

In this study, we performed a pilot analysis of data from a multicenter observational prospective study to assess the composition and over-time variation of upper airway microbiota in patients who underwent MV for non-pulmonary reasons. In particular, we analyzed upper airway microbiota at the beginning of intubation among patients intubated for non-pulmonary reasons and compared microbiota characteristics between patients who had developed VAP in the first week of ventilation and a subgroup of NO-VAP patients matched by total time of intubation. The strong relationship between lung microbiota and VAP was recently described in the study of Fenn et al., in which 55 patients suspected of VAP with a positive culture had increased dysbiosis and genus dominance compared to 55 patients with a negative culture [20].

Taking advantage of data collected in a prospective study (not yet published) aimed to assess composition and over-time variation of the upper airway microbiota in MV for non-pulmonary reasons patients, we conducted an exploratory analysis to describe upper airway microbiota characteristics of VAP and NO-VAP patients.

## Materials and methods

### Study design and setting

This exploratory analysis of data was collected in an ongoing multicenter observational prospective study (clinicaltrial.gov NCT 03720093), not yet published. Below, we briefly report the main characteristic of the above study. All consecutive mechanically ventilated patients admitted for non-pulmonary reasons and intubated for at least 48 h were enrolled and followed up to 15 days of intubation. Exclusion criteria were: patients < 18 years old, intubated for respiratory failure due to acute infectious disease (such as pneumonia). The study was approved by the research Ethics Board Brianza (no 2550, 05/07/2017). Informed consent was obtained from patients’ next-of-kin and confirmed by patients later whenever possible. This study was designed to assess the composition and over-time variation of upper airway microbiota in patients on MV for non-pulmonary reasons. Since knowledge of upper airway microbiota is limited and highly heterogenous, we decided to focus on a particular patient population not having an acute pulmonary disease, to reduce potential confounders.

Taking advantage of data collected in this study, we focus the present analysis on characteristics of upper airway microbiota in patients developing VAP, compared to those not developing VAP.

All patients who developed VAP in the first 7 days of intubation were considered in this analysis (cases cohort) and matched with a subgroup of subjects who did not develop VAP (controls cohort) by a ratio of one or, when possible, two per case. Matched controls had a mandatory criterion of comparable MV duration, equal to their matched case or at most 2 days longer, allowing to study the same time points in VAP and matched NO-VAP patients. In the matching process, antibiotic intake in the 48 h before MV was also taken into account when possible. The modified Wald method was used to calculate 95% confidence intervals (95% CI) for proportions. The optimal matching algorithm was used to identify the control group that minimizes total intra-pair dissimilarity [21].

The implementation of the algorithm in the 'optmatch' R package was used [22]. Further patient selection details are reported in Additional file 1: Fig. S1.

### Data and sampling collection

All tracheal aspirate samples collected throughout MV according to normal clinical practice were stored for each enrolled patient. In detail, tracheal aspirate collected at intubation (T0) and Day 3 (T3) were analyzed.

After pseudonymization, demographic and clinical information were stored in a web-clinical report form (web-CRF). In addition to ICU admission diagnosis, sex, age, body mass index (BMI), key medical history data, and major events that occurred in the first 15 days of intubation, including VAP, sepsis, and death, were also collected.

Diagnosis of clinical VAP has been considered according to available guidelines [23, 24] as the presence of new signs of respiratory deterioration potentially attributable to infections in association with radiological signs of pneumonia after 48 h of intubation [25]. Microbiological data were collected according to clinical practice (blood culture, microbiological cultures of tracheal aspirate or bronchoalveolar lavage). Cases of uncertainty or disagreement were reviewed independently by infectious diseases and intensivist clinicians (LA, FM and EP), and then an agreement was obtained. Sepsis was defined as life-threatening organ dysfunction measured with an increase in the Sequential [Sepsis-related] Organ Failure Assessment (SOFA) score of 2 points or more [26].

Tracheal aspirate aliquots were frozen after collection at − 80 °C without any dilution. DNA extraction was performed using QIAmp DNA Blood Kit, Qiagen®, following the user manual guide. In addition to biological samples, we included a commercial bacterial mock community (ZymoBIOMICS® HMW DNA Standard) and negative control in the analysis.

### Microbiota analysis by 16S-rRNA gene microbial profiling

Partial 16S-rRNA gene sequences were amplified from extracted DNA using primer pair Probio_Uni and/Probio_Rev, targeting the V3 region of the 16S-rRNA gene sequence [27]. 16S-rRNA gene amplification and amplicon checks were carried out as previously described [27]. 16S-rRNA gene sequencing was performed using a MiSeq (Illumina) according to a previously reported protocol [27]. The.fastq files obtained were processed using a custom script based on the QIIME2 software suite [28, 29]. Quality control retained sequences with a length between 140 and 400 bp and mean sequence quality score > 20, while sequences with homopolymers > 7 bp and mismatched primers were omitted. To calculate downstream diversity measures (alpha and beta diversity indices), 16S-rRNA Amplicon Sequence Variants (ASVs) were defined at 100% sequence homology using DADA2 [30]. ASVs not encompassing at least two sequences of the same sample were removed. All reads were classified to the lowest possible taxonomic rank using QIIME2 [28, 29] and a reference dataset from the SILVA database v. 132 [31]. Biodiversity within a given sample (alpha-diversity) was calculated through richness index (Observed ASVs) calculated for ten sub-samplings of sequenced read pools and represented by box-and-whisker plots. Similarities between samples (beta-diversity) were calculated by Bray–Curtis dissimilarity [32]. The range of similarities is calculated between values 0 and 1. PCoA (Principal Coordinates Analysis) representations of beta-diversity were performed using QIIME2 [28, 29].

### Statistical analysis

#### Clinical data

Categorical variables are presented as frequency and proportion (%), and continuous variables as median and first and last quartiles (Q1–Q3). We used χ^2^ or Fisher’s exact tests to compare categorical variables and the T-test or the Wilcoxon rank-sum test to compare continuous variables, depending on variables distribution. Values of *p* ≤ 0.05 were considered statistically significant; SAS 9.4 software (Inc., Cary, NC, USA) was used for all analyses.

#### Microbiota analysis

Differences in biodiversity between groups were assessed by T-test analysis. Furthermore, PERMANOVA analyses were performed using 1000 permutations to estimate possible significant differences among populations in PCoA analyses. Bacterial differences at the genus level were evaluated through ANOVA and Repeated Measures ANOVA using SPSS software (www.ibm.com/software/it/analytics/spss/). The post hoc analysis Tukey’s HSD (honestly significant difference) test was used for multiple comparisons. MaAsLin2 software [33] was used to determine multivariable association.

### Data availability

Raw sequences of 16S rRNA microbial profiling experiments are accessible through SRA study BioProject PRJNA708264.

## Results

Between October 2017 and March 2019, 69 patients were enrolled in two Neurological and one general Intensive Care (ICU) Units. Patients’ characteristics are reported in Additional file 1: (Fig. S1 and Table S1). In detail, the median age was 57 years (interquartile range (IQR): 39–71), 30 patients (44%) were females, and 54% were intubated for vascular diagnosis (Additional file 1: Table S2). Eighteen patients (27.5%, 95% CI 17.5–39.6) developed VAP within 15 days from intubation, of which 85% (*N* = 13) within 7 days.

Microbiota analysis was performed on samples collected from the 13 patients who developed VAP within seven days and 22 matched NO-VAP patients. Clinical characteristics of VAP and NO-VAP patients are shown in Table 1. Moreover, controls samples were selected to balance the frequency of antibiotic administration in the 48 h before the intubation. For this reason, the proportion of samples treated with antibiotics was similar in both groups (54%). Beta lactams/beta lactam inhibitors were the most frequently administered antibiotics. Eleven out of 13 VAP patients have a confirmed microbiological VAP diagnosis (tracheal aspirate positive in 9 cases, and blood cultures positive in 2 cases). In two patients, the microbiological criteria were not fulfilled. However, a review of cases confirms the clinical diagnosis of VAP. The two groups were comparable regarding gender, intubation diagnosis, and surgery at the beginning of intubation. Patients who developed VAP were younger (although only marginally significant, *p*-value = 0.06) and more frequently diagnosed with sepsis (*p*-value = 0.02). No marked differences in antibiotic administration and strategies adopted to prevent VAP during the first 72 h of intubation were found between VAP and NO-VAP patients.

**Table 1:** Patient’s characteristics and clinical events for VAP and Controls

	Total (*N* = 35)	VAP within 7 days of MV (*N* = 13^a^)	Controls NO VAP (*N* = 22)	*p-*value
Total days of intubation *(matching variable)*	7.0 (4.0–11.0)	7 (4–11)	6.5 (5–10)	0.95
Females	13 (37.1)	4 (30.8)	9 (40.9)	0.72
Age^b^	57.0 (40–67)	40.0 (34.0–58.0)	62.5 (52.0–71.0)	0.06
BMI	24.6 (21.9–26.1)	24.9 (23.2–26.0)	24.1 (21.9–26.1)	0.92
BMI ≥ 26	9 (25.0)	3 (23.1)	6 (27.2)	1.00
Vascular diagnoses at intubation^c^	18 (51.4)	5 (38.5)	13 (59.1)	0.31
GCS-Total	7.0 (4.0–10.0)	9 (5.0–11.0)	6.5 (3.0–9.0)	0.30
Any antibiotic therapy (48 h before intubation)	19 (54.3)	7 (53.9)	12 (54.6)	0.97
Surgery in the first 24 h	28 (80.0)	11 (84.6)	17 (77.3)	0.69
Tracheotomy during MV	14 (40.0)	5 (38.5)	9 (40.9)	0.87
Strategies for VAP prevention:				
Head elevation at least 30°	28 (84.9)	9 (75.0)	19 (90.5)	0.17
Suspension of sedation in the previous 24 h	17 (51.5)	8 (66.8)	9 (42.9)	0.48
Chlorhexidine oral hygiene	30 (90.9)	10 (83.3)	20 (95.2)	0.27
Subglottic aspiration (endotracheal tubes)	4 (12.2)	1 (8.3)	3 (14.5)	1.00
Any antibiotic therapy in the first 72 h of intubation	13 (37.1)^d^	6 (46.2)	9 (40.9)	0.76
Positive RX at day 3	14 (43.8)	8^e^ (66.7)	6 (30.0)	0.07
Tracheal aspirate at day 3	23 (67.7)	11 (84.6)	12 (57.1)	0.14
Positive tracheal aspirate at day 3	10 (43.5)	7^f^ (63.6)	3 (25.0)	0.10
Sepsis at day 3	6 (17.7)	5^g^ (38.5)	1 (4.8)	0.02
Death during MV	5 (14.3)	0	5 (22.7)	0.13

Data are presented as frequency and (%) or median (Q1–Q3) depending on their distribution

^a^Among the 13 VAP, seven occurred at day 3, 4 at day 5 and two at day 7 of MV

^b^When age was compared for VAP and non-VAP patients, the Wilcoxon rank-sum test *p*-value was 0.06

^c^Vascular diagnoses: 10 subarachnoid hemorrhages, six hematomas, two acute ischemic strokes. Other details on diagnoses are reported in Additional file 1: Table 2

^d^Antibiotics: amoxicillin/clavulanate (6 VAP and 3 NO-VAP); ceftriaxone (2 NO-VAP); cefazolin (2 NO-VAP); piperacillin/tazobactam (1 NO-VAP)

^e^Seven events were related to patients who developed VAP at day 3

^f^All related to VAP at day 3

^g^Four events were related to VAP on day 3

### Microbiota analysis

**Fig. 1:**
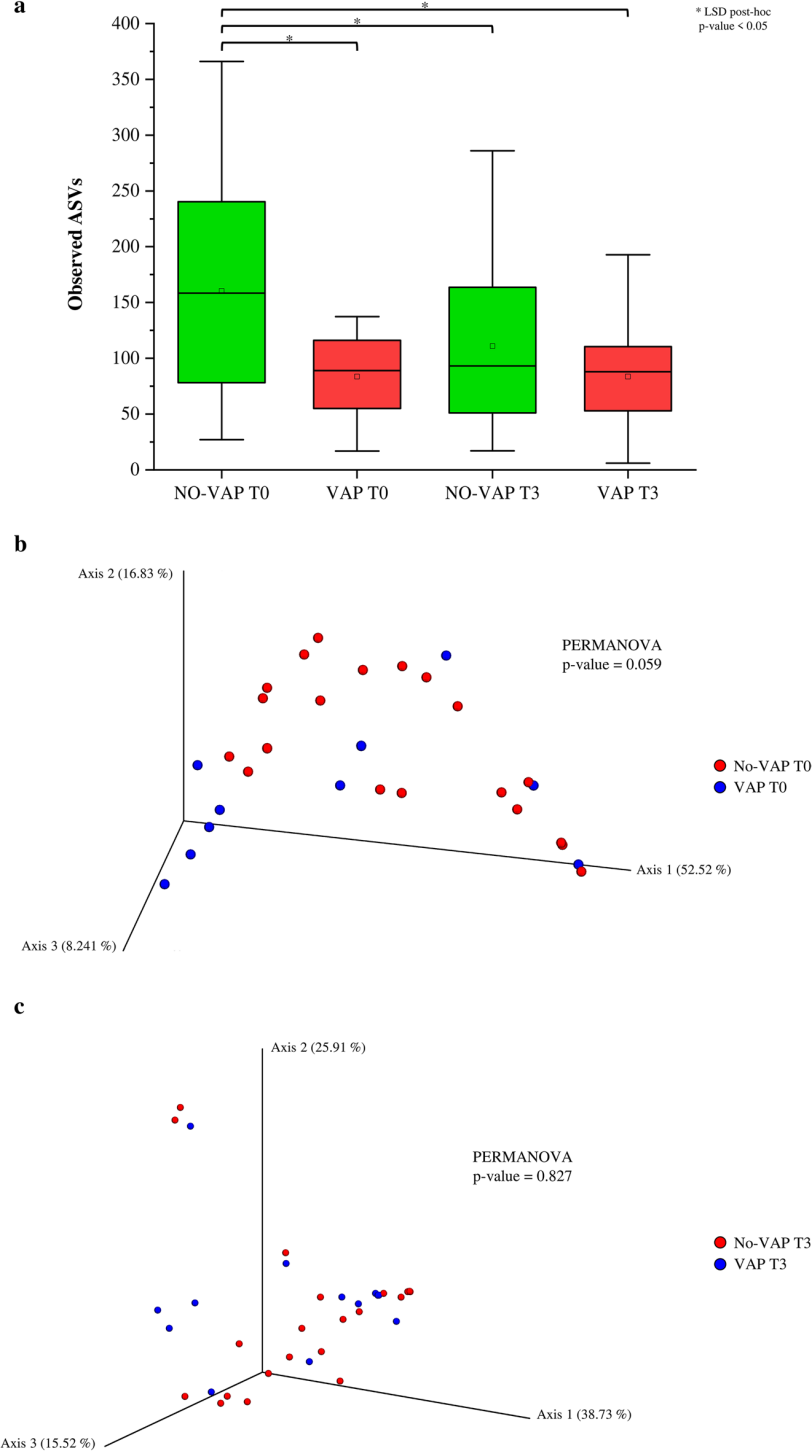
Evaluation of alpha- and beta-diversity. **a** Reports the Whiskers plot representing the richness index (based on the Amplicon Sequence Variants, ASVs) identified from VAP and NO-VAP patients over time (T0, T3). The x-axis represents the different groups, while the y-axis indicates the value of the richness index (observed Amplicon Sequence Variants, ASVs). The boxes are determined by the 25th and 75th percentiles. The whiskers are determined by 1.5 of the interquartile range. The line in the boxes represents the median, while the square represents the average. **b** Reports the principal coordinate analysis (PCoA) of the bronchial aspirate samples at T0, subdivided by VAP and NO-VAP. Panel c displays the principal coordinate analysis (PCoA) of the bronchial aspirate samples at T3, subdivided by VAP and NO-VAP

The 16S-rRNA microbial profiling analysis generated 2,644,882 sequencing reads with an average of 37,784 ± 13,540 reads per sample (Additional file 1: Table S3). Quality filtering produced a total of 2,303,978 filtered reads with an average of 32,914 ± 11,956 filtered reads per sample (Additional file 1: Table S3). In addition to biological samples, we included a commercial bacterial mock community and negative control in the analysis, to rule out possible technical and methodological contamination. To identify potential differences in the bacterial richness between VAP and NO-VAP samples at different time points, i.e., at T0 and T3, the alpha-diversity analysis based on richness index (Observed Amplicon Sequence Variants, ASVs) was performed. Intriguingly, the biodiversity analysis method allowed to identify six putative outlier samples not included in the 1.5 IQR (Inter-Quartile Range), which showed an Observed ASVs index higher than 380 (Additional file 1: Fig. S2a). These data were also confirmed by calculating the alpha diversity through Chao1 index (Additional file 1: Fig. S2a). Furthermore, the principal coordinates analysis (PCoA analysis) confirmed this hypothesis, revealing a specific cluster composed of the six putative outlier samples (Additional file 1: Fig. S2b). These six samples included both VAP and non-VAP samples at times T0 and T3 and partially represented six different patients. Therefore, we decided to exclude these six samples from our following in silico analysis, obtaining 64 samples. In addition, the PCoA analysis was used to investigate the possible impact of sepsis on microbiota composition (Figure S3), suggesting the absence of a direct relationship between this condition and the upper airway microbiota (PERMANOVA *p*-value = 0.211). Moreover, we decided to consider the samples at different times as independent to maintain a higher statistical power. In detail, the analysis of the alpha-diversity of selected samples revealed higher biodiversity of the NO-VAP group at T0 (*n* = 20, richness index 160 ± 102) compared to VAP-subjects at T0 (*n* = 10, richness index 55 ± 84) (*p*-value < 0.012) and compared to the VAP (*n* = 13, richness index 84 ± 54) and NO-VAP (*n* = 21, richness index 111 ± 72) groups at T3 (*p*-value 0.006 and *p*-value 0.043, respectively) (Fig. 1a). However, any differences were found between the VAP and NO-VAP groups at T3. Moreover, the comparison of the alpha diversity between all samples of groups T0 and T3 did not show significant differences (*p*-value = 0.098). Furthermore, analyses on possible associations between microbial biodiversity and antibiotic therapy, gender, age, and intubation diagnoses showed statistical significance (Additional file 1: Fig. S3b).

The beta-diversity analysis between VAP and NO-VAP groups at each time point suggested a slight difference in microbiota composition of the two groups at T0 (although only borderline significant: PERMANOVA *p*-value = 0.059), which disappears entirely at T3 (Fig. 1b and c). The evaluation of beta-diversity between all samples of groups T0 and T3 revealed a separate clustering of the two groups (PERMANOVA *p*-value < 0.05) (Additional file 1: Fig. S3c), although it is difficult to understand if VAP caused dysbiosis or dysbiosis caused VAP.

### Evaluation of upper airway microbiota

To evaluate the possible association between bacterial composition and time-point, antibiotic treatment and development of VAP, a multivariate analysis was performed using MaAsLin2 [33]. The analysis revealed a significant negative association of the *Streptococcus* genus at time-point T3 (*p*-value < 0.01 and *q*-value < 0.02), even if there are no particular association with the other parameters included in the analysis. This result, corroborated by the analysis of microbial biodiversity of the samples, seemed to indicate a considerable heterogeneity in microbiota compositions, suggesting the need to analyze the prevalence of each taxon to define the presumed upper airway microbiota. In detail, we considered the samples at different times as independent and we focused on the most prevalent bacterial genera represented by a prevalence > 70% in at least one group (Table 2). Only *Streptococcus* genus was present with a prevalence of > 70% and an average abundance of > 5% in all groups. Moreover, 5 bacterial genera, i.e., *Prevotella* 7, *Fusobacterium*, *Neisseria*, *Escherichia–Shigella* and *Haemophilus*, were identified with a prevalence > 70% and an average abundance > 5% in the VAP T0, NO-VAP T0 and NO-VAP T3 groups (Table 2). On the contrary, the VAP T3 group showed few bacterial taxa with a prevalence > 70%, i.e., *Streptococcus* and *Faecalibacterium* genera, confirming a high composition heterogeneity among the group samples.

**Table 2:**
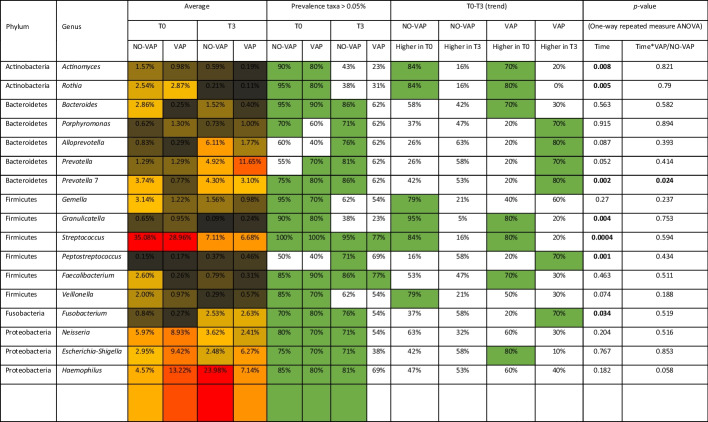
Representation of the most prevalent taxa of the study. For each genus, the average relative abundance, the prevalence for each group, and taxonomic trend are reported. Prevalences and trends > 70% are highlighted in green

To identify a specific association between VAP and NO-VAP samples and microbiota composition, we evaluated the trend of each microbial taxon identified with a prevalence > 70% (Table 2). In detail, *Actinomyces, Rothia, Granulicatella* and *Streptococcus* genera showed a greater abundance at T0 than T3 in both VAP and NO-VAP groups. Moreover, 6 genera belonging to Bacteroidetes, Firmicutes and Fusobacteria phyla, i.e., *Porphyromonas*, *Alloprevotella*, *Prevotella*, *Prevotella* 7, *Peptostreptococcus*, and *Fusobacterium*, showed a trend > 70% only in T3 VAP samples (Table 2). Moreover, a specific statistical repeated measures ANOVA analysis was performed to identify significant possible taxonomical differences. In detail, the paired analysis showed that only *Prevotella* 7 genus revealed a significant *p*-value (Table 2), presenting a generally higher relative abundance in NO-VAP samples compared to VAP.

## Discussion

The longitudinal analysis of upper airways microbiota among intubated patients for non-infectious cause of respiratory failure showed possible differences in diversity between VAP and NO-VAP samples. In detail, the alpha-diversity analysis calculated through the number of identified ASVs revealed that subjects who develop VAP during the first 7 days of intubation have a lower microbiota diversity at T0 than NO-VAP patients. The lower biodiversity of upper airways microbiota at intubation before MV in subjects developing early VAP compared to NO-VAP subjects could allow speculating that low bacterial species richness at the beginning of intubation may represent a predisposing factor to develop ventilator-associated events [34]. In addition, we corroborated the notion that upper airway microbiota undergoes a profound general reduction in species diversity during MV [15, 16, 35], which we noted both in VAP and NO-VAP groups.

The analysis of microbiota composition between samples (beta diversity) revealed a slight borderline significant difference between VAP and NO-VAP patients at T0. These results strengthen the concept of heterogeneity of lung microbiota [5, 9, 36, 37], making it particularly difficult to identify common profiles associated with VAP development, also because it is impossible to ascertain a causal effect of dysbiosis on VAP. However, lower biodiversity and MV-driven dynamism among commensal communities could promote intercurrent infectious events such as VAP.

To define the characteristic upper airway microbiota and its changes during MV, we evaluated the prevalence of each bacteria genera. The analysis over time confirmed the principal role of *Streptococcus* as commensal bacteria of the respiratory tract microbiota, confirming previous identification of positive association with healthy lungs [38, 39] and possible negative association with the development of lung disease [40]. Furthermore, some genera (*Actinomyces*, *Rothia*, *Granulicatella* and *Streptococcus*) are more represented during intubation than during ventilation. This finding indicates a possible negative association between these taxa and the duration of mechanical ventilation, allowing us to characterize better the concept of dysbiosis and dynamics of genera representation over time.

Moreover, we found that some genera (*Prevotella* 7, *Fusobacterium*, *Neisseria*, *Escherichia–Shigella* and *Haemophilus)* are present both in VAP and NO-VAP patients only at intubation, while after 3 days from the start of MV, they remain only in NO-VAP patients. These findings could suggest that early changes of a putative pulmonary core-microbiota during MV (such as with the loss of specific genera) may predispose to susceptibility to pathogens and pneumonia development. Furthermore, specific statistical repeated measures ANOVA analysis revealed a possible significant correlation between *Prevotella* 7 genus and NO-VAP condition, confirming the potential role of this bacterial genus in reducing the risk of nosocomial pneumonia [40]. In contrast, some genera (such as *Porphyromonas*, *Alloprevotella*, *Prevotella*, *Peptostreptococcus* and *Fusobacterium)* seemed to gradually increase their relative abundance in VAP samples. Consequently, they might be considered putative microbial biomarkers of this clinical event. Although *Fusobacterium* and *Porphyromonas* are common commensal bacteria of the respiratory tract, several studies reported their involvement in the development of lung disease [39, 40], suggesting a possible role in the establishment and progression of VAP. Interestingly, *Prevotella* 7 was also more abundant at T3 in VAP samples (trend > 80%), which might appear inconsistent with the above observations. These contrasting results could suggest that development of lung diseases in VAP could be related to multifactorial microbiota factors, such as alpha-diversity and bacterial composition, which are closely related.

Lastly, one of the critical aspects of microbiota analysis is considering the impact of antibiotic treatment on commensal bacteria distribution. Studies on gut microbiota confirmed that antibiotic exposure dramatically interferes with microbiota abundance and diversity [41, 42]; however, the role of antibiotics on upper airways microbiota is under investigation [16]. In our cohort, the proportion of patients with antibiotic administration within the first 72 h from intubation was similar in VAP and NO-VAP patients (about 54%). Thus, a specific multivariate analysis did not highlight the correlation between antibiotic treatment and microbiota composition, suggesting our cohort's lack of marked impact.

This study has limitations that must be addressed. First, the small sample size limits the statistical power and the sample's representativeness concerning this particular population (i.e., patients intubated for non-pulmonary reasons). This is highlighted, for example, if we consider the death proportion, which remains far different than those previously published in similar settings. In a previously published study, Emonet et al. described a mortality of 13.9% among control patients without VAP and 27.8% among VAP patients [35]. The small sample size also prevented a complete ruling out of possible confounders and an exhaustive investigation of any potential role of sepsis, antibiotics and other possible conditions which can influence the microbiota, such as the use of proton pump inhibitors, nutritional therapy, probiotics, vasopressors, opioids, non-steroidal anti-inflammatory agents. Large-scale studies are needed to corroborate the results. Second, we did not analyze oral microbiota.

Concerning the association between the gut and upper respiratory tract microbiota, little information is available to date [3, 4] and the mechanism of cross-talking between gut and lung is mostly unknown [43]. The importance of the gut–lung axis is exemplified in patients with chronic gastrointestinal diseases, such as irritable bowel syndrome (IBS) and inflammatory bowel disease (IBD), who have a higher prevalence of pulmonary diseases [44]. Only a few studies on gut–lung interaction are available in critical illness setting, demonstrating that the lung microbiome is enriched with gut bacteria both in a murine model of sepsis and in humans with established ARDS [45]. To our knowledge, no data on gut microbiota and VAP have been described.

The absence of oral samples limited us in better understanding whether the commensals found in tracheal aspirate were resident bacteria specific to the upper airway mucosa and/or were oral bacteria inhaled into the lower respiratory tract during intubation. Finally, we focused only on the characteristics of upper airway commensal associated with VAP development. Therefore, further studies are needed to consider the interactions between commensals and mucosal immunity, which plays a crucial role in regulating local homeostasis.

## Conclusion

Our analysis confirmed the negative impact of mechanical ventilation on upper airway microbiota biodiversity, highlighting a decrease in bacterial richness during the MV period in both VAP and NO-VAP patients. The collection of further clinical data and their correlation with the composition of the upper airway microbiota could have a significant translational impact in allowing the identification of the underlying clinical or pharmacological baseline components associated with the progression of dysbiosis.

## Supplementary Information

**Additional file 1: Table S1**. Characteristics of the 69 patients intubated for non-pulmonary conditions included in the main study. **Table S2.** Distribution of diagnosis at intubation in the 35 patients undergoing mechanical ventilation for non-pulmonary conditions. **Table S3.** 16S r RNA microbial profiling sequencing reads description. **Figure S1.** Study flow-chart. **Figure S2.** Evaluation of outlier samples. Panel a report the Whiskers plot representing the richness index (observed Amplicon Sequence Variants, ASVs, and Chao1 index) identified from VAP and NO-VAP patients. The x-axis represents the different groups, while the y-axis indicates the value of Observed ASVs and Chao1 indexes. The boxes are determined by the 25th and 75th percentiles. The whiskers are determined by 1.5 of interquartile range. The line in the boxes represents the median, while the square represents the average. Panel b reports the principal coordinate analysis (PCoA) of the bronchial aspirate samples, highlighting the outlier samples in blue. **Figure S3.** Evaluation of possible impact of the sepsis, antibiotic therapy, gender, age, and diagnoses on upper airway microbiota through beta- and alpha-diversity analyses. Panel a shows the principal coordinate analysis (PCoA) of the bronchial aspirate samples at T0 and T3, subdivided according to sepsis condition. Panel b investigates possible correlation between alpha diversity and antibiotic therapy, gender, age and vascular diagnoses at intubation. In detail, the y-axis of the Whiskers plot reports the richness index (based on the Amplicon Sequence Variants, ASVs), while the x-axis represents the different groups. The boxes are determined by the 25th and 75th percentiles. The whiskers are determined by 1.5 of the interquartile range. The line in the boxes represents the median, while the square represents the average. Panel c reports the principal coordinate analysis (PCoA) of the bronchial aspirate samples, subdivided by collection time, i.e., T0 and T3.

## Data Availability

The dataset of raw sequences of 16S rRNA microbial profiling experiments is available through the SRA study BioProject PRJNA708264.

## Abbreviations

MV: Mechanical ventilation
VAP: Ventilator-associated pneumonia
ICU: Intensive care unit
IQR: Interquartile range
ASVs: Amplicon Sequence Variants
PCoA: Principal coordinates analysis
Web-CRF: Web-clinical report form
BMI: Body mass index
SOFA: Sequential [Sepsis-related] Organ Failure Assessment
ARDS: Acute respiratory distress syndrome
