# Supplementary material for: Changes in upper airways microbiota in ventilator-associated pneumonia
Source: Intensive Care Med Exp. 2023 Mar 2;11:17. doi: 10.1186/s40635-023-00496-5 (PMC9981834; doi:10.1186/s40635-023-00496-5)
Supplement: Supplementary file 1 — Additional file 1: Table S1. Characteristics of the 69 patients intubated for non-pulmonary conditions included in the main study. Table S2. Distribution of diagnosis at intubation in the 35 patients undergoing mechanical ventilation for non-pulmonary conditions. Table S3. 16S r RNA microbial profiling sequencing reads description. Figure S1. Study flow-chart. Figure S2. Evaluation of outlier samples. Panel a report the Whiskers plot representing the richness index (observed Amplicon Sequence Variants, ASVs, and Chao1 index) identified from VAP and NO-VAP patients. The x-axis represents the different groups, while the y-axis indicates the value of Observed ASVs and Chao1 indexes. The boxes are determined by the 25th and 75th percentiles. The whiskers are determined by 1.5 of interquartile range. The line in the boxes represents the median, while the square represents the average. Panel b reports the principal coordinate analysis (PCoA) of the bronchial aspirate samples, highlighting the outlier samples in blue. Figure S3. Evaluation of possible impact of the sepsis, antibiotic therapy, gender, age, and diagnoses on upper airway microbiota through beta- and alpha-diversity analyses. Panel a shows the principal coordinate analysis (PCoA) of the bronchial aspirate samples at T0 and T3, subdivided according to sepsis condition. Panel b investigates possible correlation between alpha diversity and antibiotic therapy, gender, age and vascular diagnoses at intubation. In detail, the y-axis of the Whiskers plot reports the richness index (based on the Amplicon Sequence Variants, ASVs), while the x-axis represents the different groups. The boxes are determined by the 25th and 75th percentiles. The whiskers are determined by 1.5 of the interquartile range. The line in the boxes represents the median, while the square represents the average. Panel c reports the principal coordinate analysis (PCoA) of the bronchial aspirate samples, subdivided by collection time, i.e., T0 and T [file 40635_2023_496_MOESM1_ESM.docx]

**ADDITIONAL FILE**

**Figure S1.** Study flow-chart

**
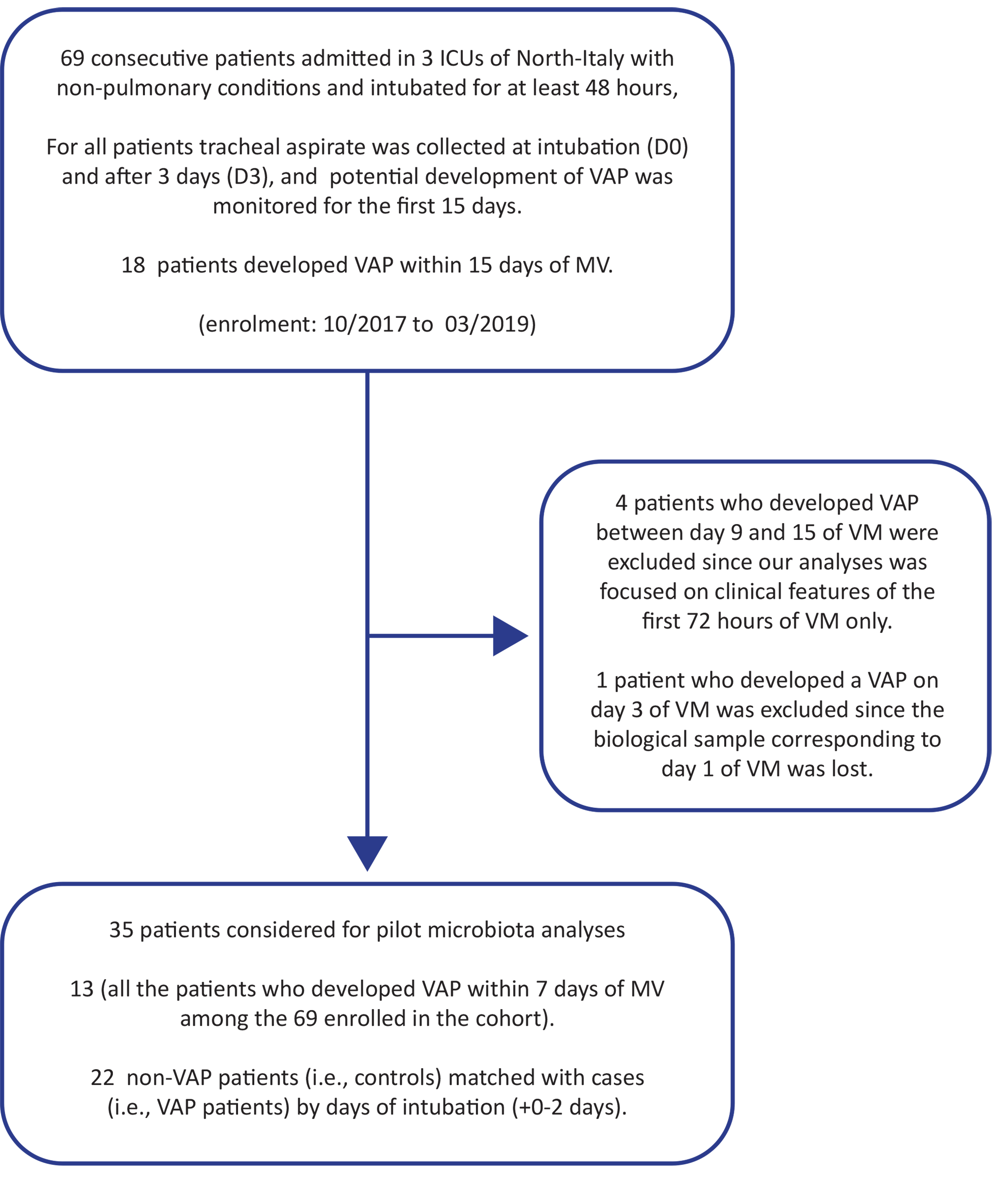
**

**Figure S2.** Evaluation of outlier samples.

**
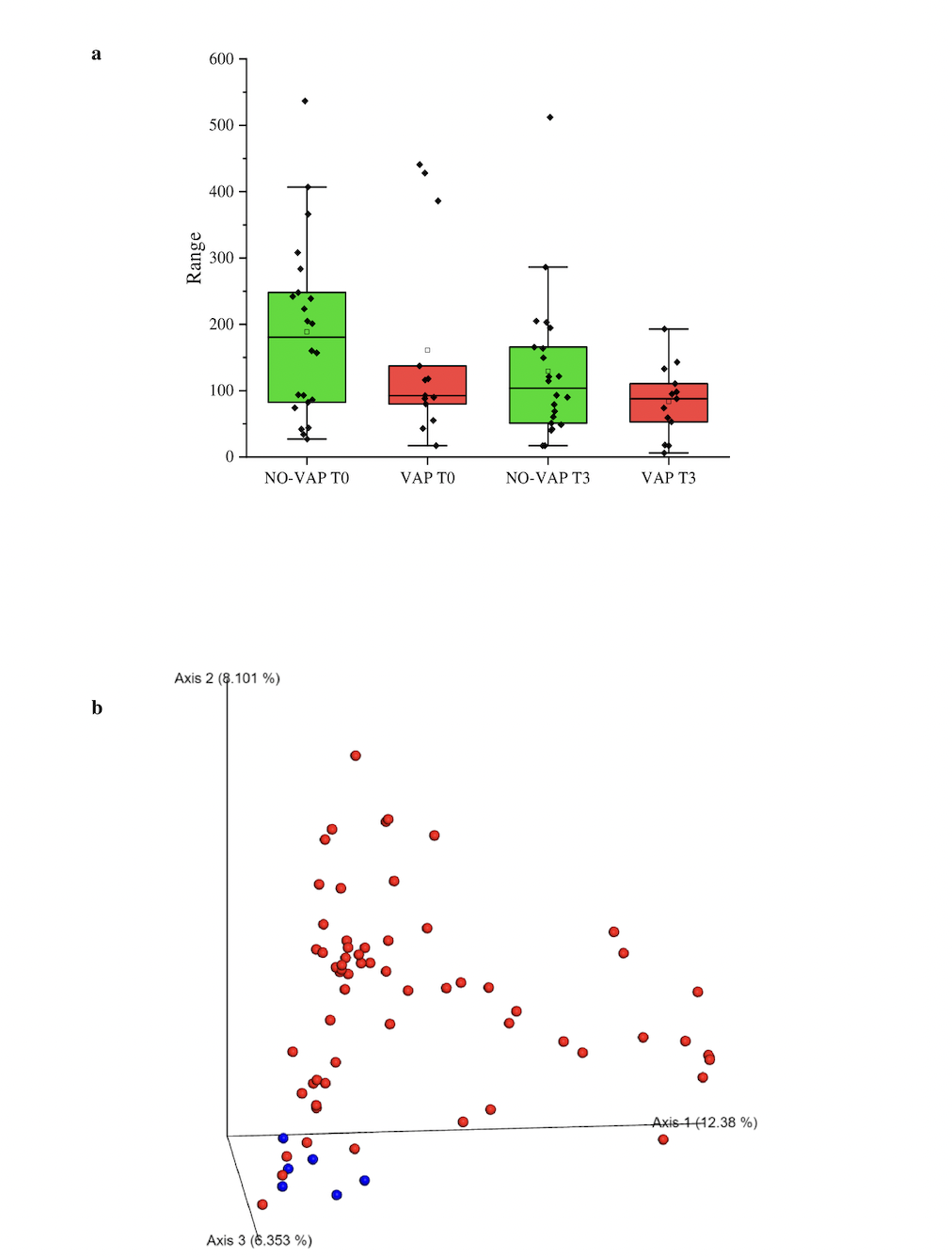
**

Panel a report the Whiskers plot representing the Observed OTUs index identified from VAP and NO-VAP patients. The x axis represents the different groups, while the y axis indicates the value of Observed OTUs index. The boxes are determined by the 25th and 75th percentiles. The whiskers are determined by 1.5 of interquartile range. The line in the boxes represented the median, while the square represents the average. Panel b reports the principal coordinate analysis (PCoA) of the bronchial aspirate samples, highlighting the outlier samples in blue.

**Figure S3.** Evaluation of possible impact of the sepsis, antibiotic therapy, gender, age, and diagnoses on upper airway microbiota through beta- and alpha-diversity analyses.

Panel a shows the principal coordinate analysis (PCoA) of the bronchial aspirate samples at T0 and T3, subdivided according to sepsis condition.

Panel b investigates possible correlation between alpha diversity and antibiotic therapy, gender, age and vascular diagnoses at intubation. In detail, the y-axis of the Whiskers plot reports the richness index (based on the Amplicon Sequence Variants, ASVs), while the x-axis represents the different groups. The boxes are determined by the 25th and 75th percentiles. The whiskers are determined by 1.5 of the interquartile range. The line in the boxes represents the median, while the square represents the average.

Panel c reports the principal coordinate analysis (PCoA) of the bronchial aspirate samples, subdivided by collection time, i.e., T0 and T3.

**Table S1**: Characteristics of the 69 patients intubated for non-pulmonary conditions included in the main study. Data are presented as frequency and (%) or median (Q1-Q3) depending on their distribution.

|  | Total cohort (N= 69) | |
| --- | --- | --- |
| Females | 30 | (43.5) |
| Age | 57 | (39-71) |
| BMI  BMI ≥ 26 | 24.5  17 | (22.7-26.2)  (28.3) |
| Vascular diagnoses at intubation | 27 | (40.9) |
| GCS-Total | 9.0 | (4.0-13.0) |
| Any antibiotic therapy within 48h before intubation | 27 | (40.9) |
| Surgery in the first 24h | 44 | (64.7) |
| Tracheotomy | 36 | (52.2) |
| VAP diagnosis within 15 days of MV | 18 | (27.5) |
| Total days of intubation | 8 | (4-15) |
| Death | 9 | (13.0) |

**Table S2**: Distribution of diagnosis at intubation in the 35 patients undergoing mechanical ventilation for non-pulmonary conditions and selected for the pilot analysis of pulmonary-microbiota composition.

| Vascular diagnoses at intubation | Diagnosis (number of patients) | | | | | |
| --- | --- | --- | --- | --- | --- | --- |
|  | Hematoma | Other*^a^* | Acute ischemic stroke | Subarachnoid hemorrhage | Traumatic brain injury | Total |
| No | 0 | 3 | 0 | 0 | 14 | 17 |
| Yes | 6 | 0 | 2 | 10 | 0 | 19 |
| Total | 6 (17.1) | 3 (8.6) | 2 (5.7) | 10 (28.6) | 14 (40.0) | 35 |

^a^ Central nervous sistem infections

**Table S3**: 16S rRNA microbial profiling sequencing reads description

|  |  | **Samples** | **Input reads** | **Filtered** | **Denoised** | **Non-chimeric** | **Final reads** |
| --- | --- | --- | --- | --- | --- | --- | --- |
| **MB01** | **T0** | **MB01-01** | 35121 | 30131 | 30131 | 29895 | 29748 |
| **MB01** | **T3** | **MB01-02** | 40907 | 35571 | 35571 | 35457 | 35359 |
| **MB02** | **T0** | **MB02-01** | 66616 | 61547 | 61547 | 54931 | 54918 |
| **MB02** | **T3** | **MB02-02** | 35314 | 31917 | 31917 | 30261 | 30244 |
| **MB03** | **T0** | **MB03-01** | 52532 | 46367 | 46367 | 45961 | 45958 |
| **MB03** | **T3** | **MB03-02** | 55017 | 46665 | 46665 | 46665 | 46663 |
| **MB05** | **T0** | **MB05-01** | 56828 | 50149 | 50149 | 49876 | 49840 |
| **MB05** | **T3** | **MB05-02** | 53366 | 47732 | 47732 | 47127 | 47127 |
| **MB06** | **T0** | **MB06-01** | 48768 | 42771 | 42771 | 42144 | 42140 |
| **MB06** | **T3** | **MB06-02** | 6008 | 4921 | 4921 | 4921 | 4470 |
| **MB15** | **T0** | **MB15-01** | 43402 | 37699 | 37699 | 32988 | 32985 |
| **MB15** | **T3** | **MB15-02** | 48566 | 42297 | 42297 | 42240 | 42213 |
| **MB17** | **T0** | **MB17-01** | 45832 | 40858 | 40858 | 39817 | 39817 |
| **MB17** | **T3** | **MB17-02** | 42285 | 37057 | 37057 | 36017 | 36005 |
| **MB22** | **T0** | **MB22-01** | 40924 | 37142 | 37142 | 36625 | 36554 |
| **MB22** | **T3** | **MB22-02** | 47343 | 41601 | 41601 | 39938 | 39934 |
| **MB24** | **T0** | **MB24-01** | 12524 | 11048 | 11048 | 10982 | 10840 |
| **MB24** | **T3** | **MB24-02** | 34253 | 28486 | 28486 | 27874 | 27740 |
| **MB25** | **T0** | **MB25-01** | 26948 | 23757 | 23757 | 23705 | 22757 |
| **MB25** | **T3** | **MB25-02** | 12140 | 10411 | 10411 | 10411 | 10125 |
| **MB26** | **T0** | **MB26-01** | 45380 | 39728 | 39728 | 36906 | 35000 |
| **MB26** | **T3** | **MB26-02** | 14367 | 12358 | 12358 | 12358 | 12225 |
| **MB27** | **T0** | **MB27-01** | 30289 | 26245 | 26245 | 26245 | 26160 |
| **MB27** | **T3** | **MB27-02** | 5717 | 4728 | 4728 | 4713 | 4559 |
| **MB29** | **T0** | **MB29-01** | 17241 | 14448 | 14448 | 14448 | 14448 |
| **MB29** | **T3** | **MB29-02** | 37750 | 32748 | 32748 | 29643 | 29631 |
| **MB37** | **T0** | **MB37-01** | 26006 | 22615 | 22615 | 22573 | 22379 |
| **MB37** | **T3** | **MB37-02** | 9914 | 8340 | 8340 | 8319 | 8109 |
| **MB38** | **T0** | **MB38-01** | 46987 | 41592 | 41592 | 41433 | 41399 |
| **MB43** | **T0** | **MB43-01** | 38792 | 34811 | 34811 | 32293 | 32287 |
| **MB43** | **T3** | **MB43-02** | 36456 | 33201 | 33201 | 32857 | 32849 |
| **MB47** | **T0** | **MB47-01** | 46860 | 40498 | 40498 | 40436 | 40398 |
| **MB47** | **T3** | **MB47-02** | 42557 | 36893 | 36893 | 36755 | 36753 |
| **Mi01-23** | **T0** | **Mi01-23-1** | 48923 | 45589 | 45589 | 43746 | 43746 |
| **Mi01-23** | **T3** | **Mi01-23-2** | 55041 | 51826 | 51826 | 47625 | 47625 |
| **Mi01-30** | **T0** | **Mi01-30-1** | 33698 | 30960 | 30960 | 28824 | 28793 |
| **Mi01-30** | **T3** | **Mi01-30-2** | 38816 | 36031 | 36031 | 34321 | 34311 |
| **PR10** | **T0** | **PR10-1** | 35946 | 33044 | 33044 | 31993 | 31990 |
| **PR10** | **T3** | **PR10-2** | 39668 | 36790 | 36790 | 35833 | 35833 |
| **PR13** | **T0** | **PR13-1** | 45018 | 42526 | 42526 | 40654 | 40654 |
| **PR13** | **T3** | **PR13-2** | 44999 | 42003 | 42003 | 41018 | 41008 |
| **PR14** | **T0** | **PR14-1** | 26095 | 24414 | 24414 | 24225 | 24146 |
| **PR14** | **T3** | **PR14-2** | 10696 | 9491 | 9491 | 9409 | 9037 |
| **PR15** | **T0** | **PR15-1** | 37610 | 35093 | 35093 | 32241 | 32234 |
| **PR15** | **T3** | **PR15-2** | 42852 | 40697 | 40697 | 38215 | 38215 |
| **PR16** | **T0** | **PR16-1** | 34465 | 32904 | 32904 | 32601 | 32495 |
| **PR16** | **T3** | **PR16-2** | 45134 | 42395 | 42395 | 38412 | 38404 |
| **PR17** | **T0** | **PR17-1** | 44899 | 42398 | 42398 | 36158 | 36154 |
| **PR17** | **T3** | **PR17-2** | 53395 | 50229 | 50229 | 49373 | 49265 |
| **PR18** | **T0** | **PR18-1** | 47382 | 43817 | 43817 | 43104 | 43104 |
| **PR18** | **T3** | **PR18-2** | 38952 | 35673 | 35673 | 34815 | 34815 |
| **PR2** | **T0** | **PR2-1** | 39867 | 36628 | 36628 | 36543 | 36507 |
| **PR2** | **T3** | **PR2-2** | 43209 | 40552 | 40552 | 38297 | 38297 |
| **PR20** | **T0** | **PR20-1** | 40195 | 37378 | 37378 | 36932 | 36916 |
| **PR20** | **T3** | **PR20-2** | 42245 | 39259 | 39259 | 39165 | 39165 |
| **PR21** | **T0** | **PR21-1** | 51348 | 48103 | 48103 | 47155 | 46994 |
| **PR21** | **T3** | **PR21-2** | 24957 | 23203 | 23203 | 22774 | 22630 |
| **PR22** | **T0** | **PR22-1** | 38325 | 36593 | 36593 | 35110 | 35110 |
| **PR22** | **T3** | **PR22-2** | 41035 | 38929 | 38929 | 38929 | 38921 |
| **PR3** | **T0** | **PR3-1** | 50318 | 46786 | 46786 | 41716 | 41704 |
| **PR3** | **T3** | **PR3-2** | 42952 | 40476 | 40476 | 39676 | 39669 |
| **PR4** | **T0** | **PR4-1** | 14927 | 13673 | 13673 | 13114 | 13049 |
| **PR4** | **T3** | **PR4-2** | 24503 | 22834 | 22834 | 20778 | 20745 |
| **PR5** | **T0** | **PR5-1** | 34910 | 32766 | 32766 | 32448 | 32388 |
| **PR5** | **T3** | **PR5-2** | 48506 | 45977 | 45977 | 42440 | 42413 |
| **PR6** | **T0** | **PR6-1** | 51159 | 48145 | 48145 | 46928 | 46804 |
| **PR6** | **T3** | **PR6-2** | 44959 | 42167 | 42167 | 38075 | 38075 |
| **PR9** | **T0** | **PR9-1** | 38985 | 35746 | 35746 | 30727 | 30720 |
| **PR9** | **T3** | **PR9-2** | 39789 | 36950 | 36950 | 35105 | 35105 |
